# 

**Published:** 1887-02

**Authors:** 


### TABLE OF CONTENTS.

### Original and Selected Articles.

The Duties of Medical Men to Medical Societies, by Virgil O. Hardon, M.D., Atlanta, Ga.. 45
Report of a Case of Diphtheria with Rare Complications, by J. A. Green, of Georgia........... 53
Ligation of the Femoral Artery in a Case of Femoro-Popliteal Aneurism, by J. McF. Gaston, M.D.......... 57
The English System of Diet for Nervous Exhaustion .......... 61
Gastrotomy for the Removal of a Swallowed Knife. Recovery of the Patient. With Illustration, by Augustus C. Bernays, M. D.. Louisville, Ky.......... 63

### Abstracts and Gleanings.

Letter from Germany.......... 66
Hot Bath.......... 68
Solanine—A Substitute for Morphine.......... 69
Fractured Femur.......... 70
Suprapubic Cystotomy.......... 71
Digestion of Infants.......... 72
Quinine in Whooping Cough, Hemorrhoids..... 74
On Vaginal Extirpation of the Uterus; New Method of Employing Electricity in Neuralgia .......... 75
Burns; Treatment of Bright's Disease.......... 76

### Scientific Items.

The Resistance of the Atmosphere; The Purity of Mid-Atlantic Air.......... 77
Electric Lamp; Impure Ice; Scientific Fish Story. .......... 78

### Practical Notes and Formulæ.

Whooping Cough; For Vomiting; Elixir of Iron Pyrophosphate, Quinine and Strychnine ..... 79

Eulylyp'ol; Use of Lanoline in Midwifery Practice; Biniodide of Mercury in Diphtheria and Scarlatina; Conjunctivitis.......... 80

For Headache of Dysmenorrhœa; Tonic for Children; Tonic in Phthisis; Chronic Diarrhœa; Chronic Skin Eruptions; Infantile Colic; Chronic Bronchitis.......... 8[illegible]

### Editorials and Miscellaneous.

Editorial Brevities.......... 8[illegible]

Dr. Powell to go to the International Congress; Death of the Managing Editor's Son.......... 8[illegible]

Support Your Home Journals; War Remedies; 

